# Classification of *Epidermal Growth Factor Receptor* Gene Mutation Status Using Serum Proteomic Profiling Predicts Tumor Response in Patients with Stage IIIB or IV Non-Small-Cell Lung Cancer

**DOI:** 10.1371/journal.pone.0128970

**Published:** 2015-06-05

**Authors:** Lin Yang, Chuanhao Tang, Bin Xu, Weixia Wang, Jianjie Li, Xiaoyan Li, Haifeng Qin, Hongjun Gao, Kun He, Santai Song, Xiaoqing Liu

**Affiliations:** 1 Department of Pulmonary Oncology, 307 Hospital, PLA, Beijing, China; 2 National Center of Biomedical Analysis, Beijing, China; 3 Cancer Center, 307 Hospital, PLA, Beijing, China; University of Algarve, PORTUGAL

## Abstract

**Objectives:**

*Epidermal growth factor receptor (EGFR)* gene mutations in tumors predict tumor response to EGFR tyrosine kinase inhibitors (EGFR-TKIs) in non-small-cell lung cancer (NSCLC). However, obtaining tumor tissue for mutation analysis is challenging. Here, we aimed to detect serum peptides/proteins associated with *EGFR* gene mutation status, and test whether a classification algorithm based on serum proteomic profiling could be developed to analyze *EGFR* gene mutation status to aid therapeutic decision-making.

**Patients and Methods:**

Serum collected from 223 stage IIIB or IV NSCLC patients with known *EGFR* gene mutation status in their tumors prior to therapy was analyzed by matrix-assisted laser desorption/ionization time-of-flight mass spectrometry (MALDI-TOF-MS) and ClinProTools software. Differences in serum peptides/proteins between patients with *EGFR* gene TKI-sensitive mutations and wild-type *EGFR* genes were detected in a training group of 100 patients; based on this analysis, a serum proteomic classification algorithm was developed to classify *EGFR* gene mutation status and tested in an independent validation group of 123 patients. The correlation between *EGFR* gene mutation status, as identified with the serum proteomic classifier and response to EGFR-TKIs was analyzed.

**Results:**

Nine peptide/protein peaks were significantly different between NSCLC patients with *EGFR* gene TKI-sensitive mutations and wild-type *EGFR* genes in the training group. A genetic algorithm model consisting of five peptides/proteins (m/z 4092.4, 4585.05, 1365.1, 4643.49 and 4438.43) was developed from the training group to separate patients with *EGFR* gene TKI-sensitive mutations and wild-type *EGFR* genes. The classifier exhibited a sensitivity of 84.6% and a specificity of 77.5% in the validation group. In the 81 patients from the validation group treated with EGFR-TKIs, 28 (59.6%) of 47 patients whose matched samples were labeled as “mutant” by the classifier and 3 (8.8%) of 34 patients whose matched samples were labeled as “wild” achieved an objective response (p<0.0001). Patients whose matched samples were labeled as “mutant” by the classifier had a significantly longer progression-free survival (PFS) than patients whose matched samples were labeled as “wild” (p=0.001).

**Conclusion:**

Peptides/proteins related to *EGFR* gene mutation status were found in the serum. Classification of *EGFR* gene mutation status using the serum proteomic classifier established in the present study in patients with stage IIIB or IV NSCLC is feasible and may predict tumor response to EGFR-TKIs.

## Introduction

Lung cancer is the leading cause of cancer-related death worldwide [1]. Non-small-cell lung cancer (NSCLC) is the most common histologic type of the disease and accounts for approximately 80% of lung cancers [2]. Because more than 70% of patients with lung cancer are diagnosed with advanced-stage disease [3], systemic treatment plays an important role in clinical management. Chemotherapy has been the cornerstone of treatment for NSCLC for many years. However, epidermal growth factor receptor tyrosine kinase inhibitors (EGFR-TKIs), such as erlotinib, gefitinib and icotinib, have been shown to greatly improve clinical outcomes and safety when compared with chemotherapy in some patients with advanced NSCLC [4–8]. EGFR-TKI sensitivity has been associated with activating mutations in the kinase domain of the *EGFR* gene, especially an *exon 19* deletion and mutations in *exon 21(L858R)* and *exon 18(G719X)* [9–11]. All *EGFR* gene TKI-sensitive mutations result in activation of the EGFR tyrosine kinase domain, which is the target of EGFR-TKIs. Therefore, patients with these *EGFR* gene TKI-sensitive mutations have a significantly better response to EGFR-TKIs, whereas those with wild-type *EGFR* genes exhibit a worse tumor response. Assessment of *EGFR* gene mutation status is critically important for therapeutic decision-making.

National comprehensive cancer network (NCCN) guidelines state that DNA mutational analysis in tumor cells is the preferred method to assess *EGFR* gene mutation status. However, in some cases, tumor tissue either is inadequate for molecular testing because of its small quantity or very low tumor content or is not readily available [3]. Several groups have detected *EGFR* gene mutations in DNA isolated from plasma [3, 12–16] or serum samples [17, 18], which serve as substitutes for tumor tissue; some groups have demonstrated a correlation between mutation status in the plasma/serum and tumor tissue [3, 12, 13, 15–18]. Furthermore, *EGFR* gene mutations detected in plasma or serum may be predictive of the response to EGFR-TKIs [3, 13, 14, 16, 18]. However, the methods used to assess *EGFR* gene mutation status in plasma or serum samples are not approved by the current guidelines. Thus, other sensitive and noninvasive approaches for evaluating *EGFR* gene mutation status using surrogate tumor tissues to predict EGFR-TKI efficacy are still needed.

Matrix-assisted laser desorption/ionization time-of-flight mass spectrometry (MALDI-TOF-MS) is a sensitive, rapid, inexpensive, and simple technique for proteomic analysis of complex biological samples, such as tissue, urine and blood [19–26]. Peaks in the mass spectrum correspond to ions formed from relatively abundant species in the sample, predominantly peptides and proteins. Recently, peptide mass fingerprinting based on MALDI-TOF-MS has been widely used to detect diagnostic, prognostic, and predictive proteomic biomarkers. In recently published studies, peptide mass fingerprinting has been successfully applied to analyze serum from patients and healthy controls to detect differences in peptides/proteins; these differences were used to develop classification algorithms for disease diagnosis [22–25]. In addition, peptide mass fingerprinting can detect differences in serum/plasma peptides/proteins between subgroups of patients with same type of disease. Taguchi [26] and Wu [27] used MALDI-TOF-MS to analyze serum and plasma from NSCLC patients; they observed subtle differences in serum/plasma peptides/proteins between two subgroups that experienced significantly different EGFR-TKI efficacies and developed classification algorithms using differential peptides/proteins to predict the efficacy of EGFR-TKI in NSCLC patients. Because the efficacy of EGFR-TKI has been associated with *EGFR* gene mutation status, the constituting peptides/proteins of the serum/plasma classification algorithms developed by Taguchi and Wu to predict EGFR-TKI efficacy may be associated with *EGFR* gene mutation status [27, 28].

In this study, we aimed to detect serum peptides/proteins associated with *EGFR* gene mutation status and test whether a classification algorithm based on serum proteomic profiling could be developed for analysis of *EGFR* gene mutation status to assist in therapeutic decision-making. To accomplish this, we applied peptide mass fingerprinting using MALDI-TOF-MS coupled with ClinProTools software to analyze serum from 223 NSCLC patients with a known *EGFR* gene mutation status (i.e., determined by amplification refractory mutation system [ARMS] in tumor tissue) and detect differences in serum peptides/proteins between NSCLC patients with *EGFR* gene TKI-sensitive mutations and NSCLC patients with wild-type *EGFR* genes. We developed a serum proteomic classifier to evaluate *EGFR* gene mutation status and tested the classifier on an independent validation group. We also analyzed correlations between *EGFR* gene mutation status as identified by the serum proteomic classifier and response to EGFR-TKIs to test the potential utility of *EGFR* gene mutation status identified by the serum proteomic classifier for predicting clinical responses to EGFR-TKI treatment.

## Patients and Methods

### Patients and samples

To be eligible for the study, patients were required to have pathologically confirmed stage IIIB or IV NSCLC, an Eastern Cooperative Oncology Group performance status of 0 to 2, predefined *EGFR* gene mutation status in tumor tissues based on ARMS (scorpions amplification refractory mutation system, Qiagen, Germany) prior to therapy, and available serum. Only patients treated at 307 Hospital of PLA from May 2011 to April 2013 were enrolled. This study was performed according to protocols approved by the local ethical committee (the Ethics Committees of 307 Hospital, PLA), and all the patients provided written informed consent to participate in this study and gave permission for the use of their blood samples. For the tumor response assessment, we evaluated objective responses after 8 weeks of treatment on the basis of computed tomography (CT) scans. Tumor response was determined according to RECIST 1.0. Overall survival (OS) was defined as the time from the date of lung cancer diagnosis to the date of death. Progression free survival (PFS) was defined as the time from the start of EGFR-TKI treatment to the date of disease progression or death from any cause. The cutoff date for follow-up was November 10, 2014. Smoking status was based on records from the patients’ first clinic visits, and people who had smoked more than 100 cigarettes in their lifetime were considered smokers. Laboratory data were obtained and recorded independently by investigators who were blinded to the clinical data until the analyses were completed by a biostatistician.

Fifty patients were randomly selected from patients with *EGFR* gene TKI-sensitive mutations and wild-type *EGFR* genes respectively (a total of 100 patients) to form the training group for the detection of differences in serum peptides/proteins between NSCLC patients with *EGFR* gene TKI-sensitive mutations and NSCLC patients with wild-type *EGFR* genes, and the generation of the classification model, and the remaining patients formed the validation group to test the model.

The patients fasted overnight. All blood samples were collected before the patients received first-line treatment. Blood samples were collected in vacuum blood collection tubes containing coagulant and separation gel and centrifuged at 3000 rpm for 10 min at 4°C to separate the serum. The supernatant was divided into 100-μl aliquots and stored at −80°C until processing.

### Peptidome isolation

Serum samples were thawed on ice and fractionated with weak cation exchange magnetic beads (MB-WCX, National Center of Biomedical Analysis, China). The samples were processed following three steps: binding, washing and elution. For each analysis, 5 μl of beads washed three times in 50 μl of binding solution (National Center of Biomedical Analysis, China), 20 μl of binding solution and 5 μl of sample were added into an Eppendorf tube and incubated for 10 min at room temperature. The tube was placed on a magnetic bead separation device to isolate the peptidome. The supernatant was removed, and the beads were washed three times with 100 μl of washing solution (National Center of Biomedical Analysis, China) to discard unbound proteins. Finally, the beads were washed with 20 μl of eluting solution (National Center of Biomedical Analysis, China) to acquire bound proteins for MALDI-TOF-MS analysis.

### MALDI-TOF-MS analysis

For the MALDI-TOF-MS analysis, 1 μl of peptide eluate mixed 1:1(v/v) with a matrix solution consisting of saturated α-cyano-4-hydroxy-cinnamic acid (α-HCCA, Bruker Daltonics, Germany) in 50% acetonitrile (ACN, Sigma-Aldrich, USA) and 0.1% trifluoroacetic acid (TFA, Sigma-Aldrich, USA) was spotted onto the sample anchor spots of an AnchorChip 600/384 target plate (Bruker Daltonics, Germany) and allowed to air-dry at room temperature to let the matrix crystallize. ClinProt Peptide Calibration Standard I (Bruker Daltonics, Germany), a commercially available mixture of protein/peptide calibrators that consisted of angiotensin II (m/z 1,047.19), angiotensin I (m/z 1,297.49), substance P (m/z 1,348.64), bombesin (m/z 1,620.86), ACTH clip 1–17 (m/z 2,094.43), ACTH clip 18–39 (m/z 2,466.48), and somatostatin (m/z 3,149.57) was mixed 1:1 (v/v) with matrix solution, and 0.5 ml was deposited onto calibrant anchor spots of the AnchorChip target plate for instrument calibration.

Mass spectrometry analyses were performed on an Ultraflex III MALDI-TOF-MS (Bruker Daltonics, Germany). The operating conditions were as follows: linear positive ion mode; repetition rate, 200 Hz; ion source voltages, 25 and 23.50 kV; lens voltage, 6.5 kV; pulsed ion extraction time, 100 ns. For matrix suppression, we used a high gating factor with signal suppression of up to 300 m/z. For each spectrum, 3000 shots were acquired manually from six random positions over the surface of the spot (i.e., 500 shots per position). Data acquisition was carried out at 43% of the maximum laser energy. Each spectrum was externally calibrated. Peaks in the m/z range of 800–10,000 Da were recorded with the FlexControl acquisition software v3.4 (Bruker Daltonics, Germany).

### Bioinformatics

#### Spectral processing

ClinProTools software v2.1 (Bruker Daltonics, Germany) was used to automatically process MALDI-TOFMS spectra data using data preparation settings according to the following standard workflow: Each raw spectrum was normalized to its total ion current; all the spectra were recalibrated using the prominent, common m/z values; baseline subtraction, smoothing, and peak detection were performed; and peak areas for each spectrum were calculated. The signal-to-noise ratio was set at 5 for peak detection. Peak areas were calculated using zero level integration type. Spectra were also ‘‘top hat” baseline subtracted with the minimum baseline width set to 10%, smoothed and processed in the 800–10,000 Da range.

#### Training and classification model establishment in the training group

Only spectra from the training group were used. Differences in peptide peaks between patients with *EGFR* gene TKI-sensitive mutations and patients with wild-type *EGFR* genes were selected using peak areas on the basis of statistical differences. Built-in mathematical models in ClinProTools 2.1 (i.e., genetic algorithm (GA), supervised neural network (SNN) algorithm and quick classifier (QC) algorithm) were then used to select peptide peaks and set up classification models to determine the optimal separation planes between samples from patients with *EGFR* gene TKI-sensitive mutations and wild-type *EGFR* genes. After each model was generated, a random cross-validation process was carried out with the software, and the percent to leave out and number of iterations were set at 20 and 10, respectively.

To determine the accuracy of the class prediction model, the software quantifies cross-validation and recognition capability. Cross-validation is a measure of the reliability of a model and can be used to predict how a model will behave in the future. This method is used for evaluating the performance of an algorithm for a given data set and under a given parameterization. Recognition capability describes the performance of an algorithm, i.e., the proper classification of a given data set.

#### Blind test of the classification model that most efficiently separated samples from patients with *EGFR* gene TKI-sensitive mutations from samples from patients with wild-type *EGFR* genes in the validation group

This validation was performed in a blinded manner in that MALDI-TOF-MS analysis was performed and samples were classified before the clinical outcome data were made available to the investigators.

For each patient from the validation groups, a corresponding spectrum was presented to the selected classification model (named classifier), which then returned a label, either “mutant” (i.e., classification to class consisting of samples from patients with *EGFR* gene TKI-sensitive mutations) or “wild” (i.e., classification to class consisting of samples from patients with wild-type *EGFR* gene), or output a message that the spectrum was unclassifiable. The results from the selected classification model were compared with findings from ARMS in tumors to estimate the separation efficiency of the model.

### Statistical analysis

The clinical and disease characteristics between different arms, the objective response rate (ORR) and disease control rate (DCR) between patients whose matched samples were labeled as “mutant” and “wild” were compared using a χ^2^ or Fisher’s exact test. The concordance between ARMS in tumors and the serum proteomic classifier in evaluating *EGFR* gene mutation status was assessed using a Kappa test. Survival curves were estimated by the Kaplan—Meier method, and differences between curves were evaluated by the log-rank test. Statistical analyses were performed with SPSS software, v19.0 (SPSS Inc., USA). A p-value less than 0.05 was considered statistically significant.

## Results

### Patient Characteristics

A total of 223 patients met the enrollment criteria and were enrolled in this study. Based on the criterion of ARMS in tumors, there were 102 patients with *EGFR* gene TKI-sensitive mutations and 121 patients with wild-type *EGFR* genes. Fifty patients were randomly selected from those with *EGFR* gene TKI-sensitive mutations and from those with wild-type *EGFR* genes (i.e., a total of 100 patients) to form the training group, and the remaining 123 patients (i.e., 52 patients with *EGFR* gene TKI-sensitive mutations and 71 with wild-type *EGFR* genes) formed the validation group. The clinical and disease characteristics of all the patients are listed in Table 1. The patients were balanced between the training group and the validation group (Table 2). In the training group, there were no significant differences between patients with *EGFR* gene TKI-sensitive mutations and wild-type *EGFR* genes with respect to age, histologic type, or disease stage, but differences in sex and smoking history were observed between these two arms, with more females and more non-smokers in patients with *EGFR* gene TKI-sensitive mutations (Table 3).

**Table 1:** Clinical and disease characteristics of all patients.

Characteristics	No. of patients (N = 223)	% of patients
**Age, years**		
** Mean**	57.0	
** Standard deviation**	11.5	
**Sex**		
** Male**	109	48.9
** Female**	114	51.1
**Smoking history**		
** Smoker**	95	42.6
** Never smoker**	128	57.4
**Histologic type**		
** ADC**	205	91.9
** SCC**	13	5.8
** Other**	5	2.3
**Disease stage**		
** IIIB**	41	18.4
** IV**	182	81.6
**EGFR-TKI treatment**		
** No**	69	30.9
** Yes**	154	69.1
** First-line**	72	32.3
** Second-line**	61	27.4
** Third-line or greater**	21	9.4
***EGFR* gene mutation status determined by ARMS**		
***E19del***	55	24.7
***L858R***	43	19.3
***G719X***	4	1.8
***wild-type***	121	54.2

ADC = adenocarcinoma; SCC = squamous cell carcinoma; TKI = tyrosine kinase inhibitor; EGFR = epidermal growth factor receptor; ARMS = amplification refractory mutation system; *E19del* = *exon 19* deletion; *L858R* = *exon 21 (L858R)* mutation; *G719X* = *exon 18 (G719X)* mutation.

**Table 2:** Clinical and disease characteristics of patients in the training and validation groups.

Characteristics	Training group (N = 100)	Validation group (N = 123)	P value
**Age, y**			0.155
** Mean**	58.2	56.0	
** Standard deviation**	11.1	11.7	
**Sex, No. (%)**			0.813
** Male**	48(48.0)	61(49.6)	
** Female**	52(52.0)	62(50.4)	
**Smoking history, No. (%)**			0.870
** Smoker**	42(42.0)	53(43.1)	
** Never smoker**	58(58.0)	70(56.9)	
**Histologic type, No. (%)**			0.868
** ADC**	93(93.0)	112(91.1)	
** SCC**	5(5.0)	8(6.5)	
** Others**	2(2.0)	3(2.4)	
**Disease stage, No. (%)**			0.893
** IIIB**	18(18.0)	23(18.7)	
** IV**	82(82.0)	100(81.3)	
**EGFR-TKI treatment, No. (%)**			ND
** No**	29(29.0)	40(32.5)	
** Yes**	71(71.0)	83(67.5)	
** First-line**	34(34.0)	38(30.9)	
** Second-line**	28(28.0)	33(26.8)	
** Third-line or greater**	9(9.0)	12(9.8)	
***EGFR* gene mutation status determined by ARMS, No. (%)**			ND
***E19del***	27(27.0)	28(22.8)	
***L858R***	21(21.0)	22(17.9)	
***G719X***	2(2.0)	2(1.6)	
***wild-type***	50(50.0)	71(57.7)	

ND = not done.

**Table 3:** Clinical and disease characteristics of patients with EGFR gene TKI-sensitive mutations and patients with a wild-type EGFR gene in the training group.

Characteristics	Mutation arm (N = 50)	Wild-type arm (N = 50)	P value
**Age, y**			0.419
** Mean**	57.3	59.1	
** Standard deviation**	11.6	10.5	
**Sex, No. (%)**			0.016
** Male**	18(36.0)	30(60.0)	
** Female**	32(64.0)	20(40.0)	
**Smoking history, No. (%)**			0.043
** Smoker**	16(32.0)	26(52.0)	
** Never smoker**	34(68.0)	24(48.0)	
**Histologic type, No. (%)**			0.131
** ADC**	49(98.0)	44(88.0)	
** SCC**	1(2.0)	4(8.0)	
** Others**	0(0)	2(4.0)	
**Disease stage, No. (%)**			0.603
** IIIB**	8(16.0)	10(20.0)	
** IV**	42(84.0)	40(80.0)	
**EGFR-TKI treatment, No. (%)**			ND
** No**	3(6.0)	26(52.0)	
** Yes**	47(94.0)	24(48.0)	
** First-line**	29(58.0)	5(10.0)	
** Second-line**	15(30.0)	13(26.0)	
** Third-line or greater**	3(6.0)	6(12.0)	
***EGFR* gene mutation status determined by ARMS, No. (%)**			ND
***E19del***	27(54.0)	0(0)	
***L858R***	21(42.0)	0(0)	
***G719X***	2(4.0)	0(0)	
***wild-type***	0(0)	50(100)	

### Differences of peaks in serum between patients with *EGFR* gene TKI-sensitive mutations and patients with wild-type *EGFR* genes in the training group

**Fig 1:**
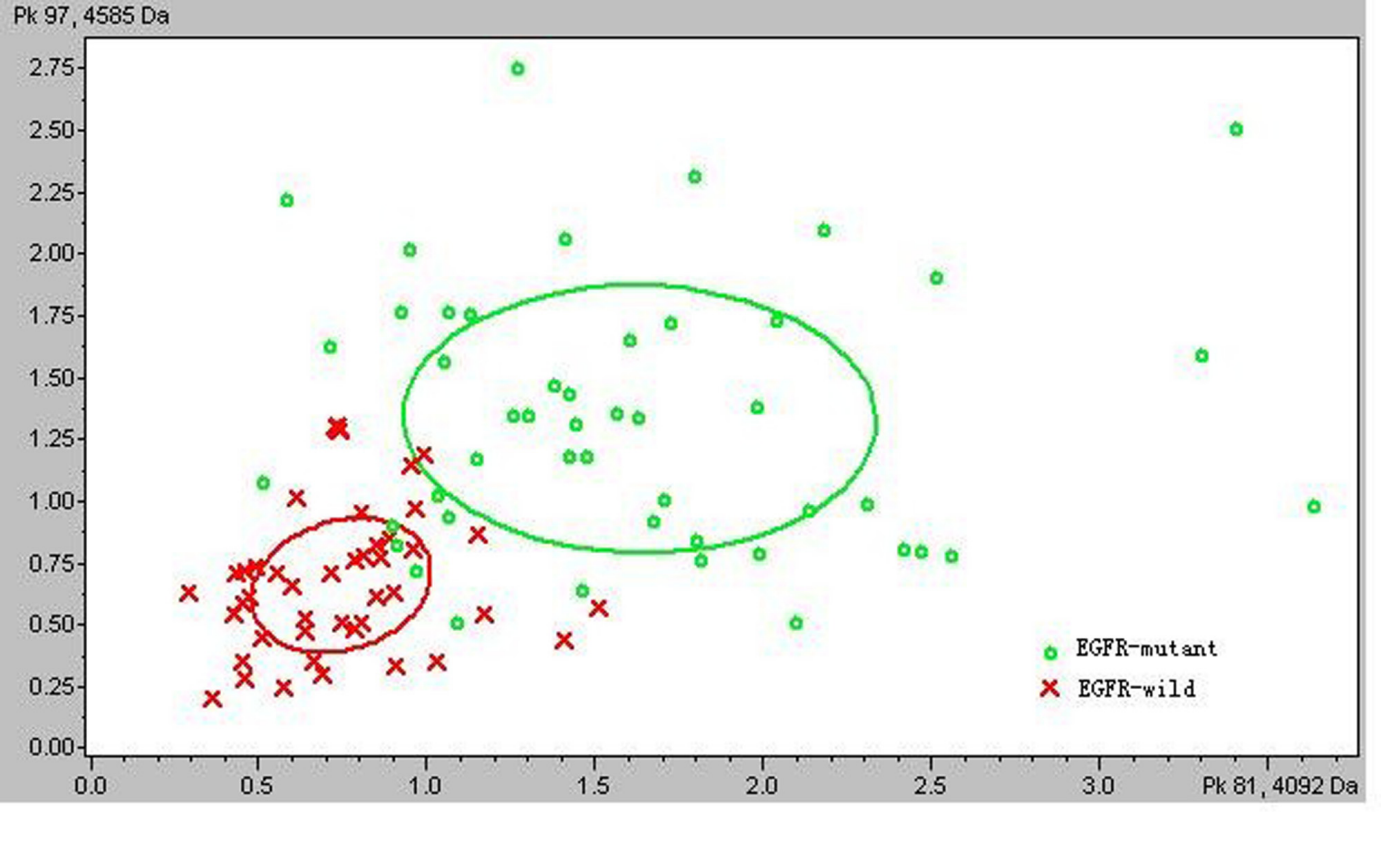
2D peak distribution of peptides with m/z 4092.4 (x-axis) and 4585.05 (y-axis) between patients with *EGFR* gene TKI-sensitive mutations (green circles) and patients with wild-type *EGFR* genes (red crosses). The discriminating features of the two selected peptides were generated by ClinProTools bioinformatics software. The values represent the peptide abundance ratio, and these values were significantly different between patients with *EGFR* gene TKI-sensitive mutations and patients with wild-type *EGFR* genes. The ellipses represent the standard deviation of the class average of the peak areas/intensities.

A total of 129 peptide peaks were identified in the spectra of the training group data set generated by MALDI-TOF-MS, and 9 peaks were significantly different (p<0.05) between the patients with *EGFR* gene TKI-sensitive mutations and patients with wild-type *EGFR* genes (Table 4). Two signals (with m/z 1365.1 and 1866.47) exhibited a lower peak area and seven signals (with m/z 3315.75, 3883.79, 3956.66, 4092.4, 4585.05, 4643.49, and 5866.96) exhibited a higher peak area in patients with *EGFR* gene TKI-sensitive mutations compared to patients with wild-type *EGFR* genes. Peptide peaks with m/z 4092.4 and 4585.05 exhibited the greatest difference in peak areas between patients with *EGFR* gene TKI-sensitive mutations and patients with wild-type *EGFR* genes (p<0.00001). Therefore, these two peaks (m/z 4092.4, x axis; m/z 4585.05, y axis) were plotted in a 2D peak distribution view (Fig 1).

**Table 4:** The 9 differential peaks in serum from patients with *EGFR* gene TKI-sensitive mutations and patients with wild-type *EGFR* genes in the training group.

m/z	Peak areas of the wild-type arm (X±S)	Peak areas of the mutation arm (X±S)	P value
**Signals showed a lower peak area in patients with *EGFR* gene TKI-sensitive mutations**			
**1365.1**	15.96±5.37	9.1±4.01	< 0.000001
**1866.47**	638.6±548.7	170.42±124.03	0.00393
**Signals showed a higher peak area in patients with *EGFR* gene TKI-sensitive mutations**			
**3315.75**	40.92±26.8	77.98±59.56	0.000608
**3883.79**	7.13±2.97	12.26±5.33	0.00000221
**3956.66**	32.42±31.73	56.98±35.78	0.00163
**4092.4**	4.57±1.63	10±4.29	< 0.000001
**4585.05**	4.04±1.67	8.15±3.3	< 0.000001
**4643.49**	30.23±14.08	48.54±23.42	0.0000792
**5866.96**	1.8±0.97	3.96±3.18	0.0201

### Classification model establishment

**Fig 2:**
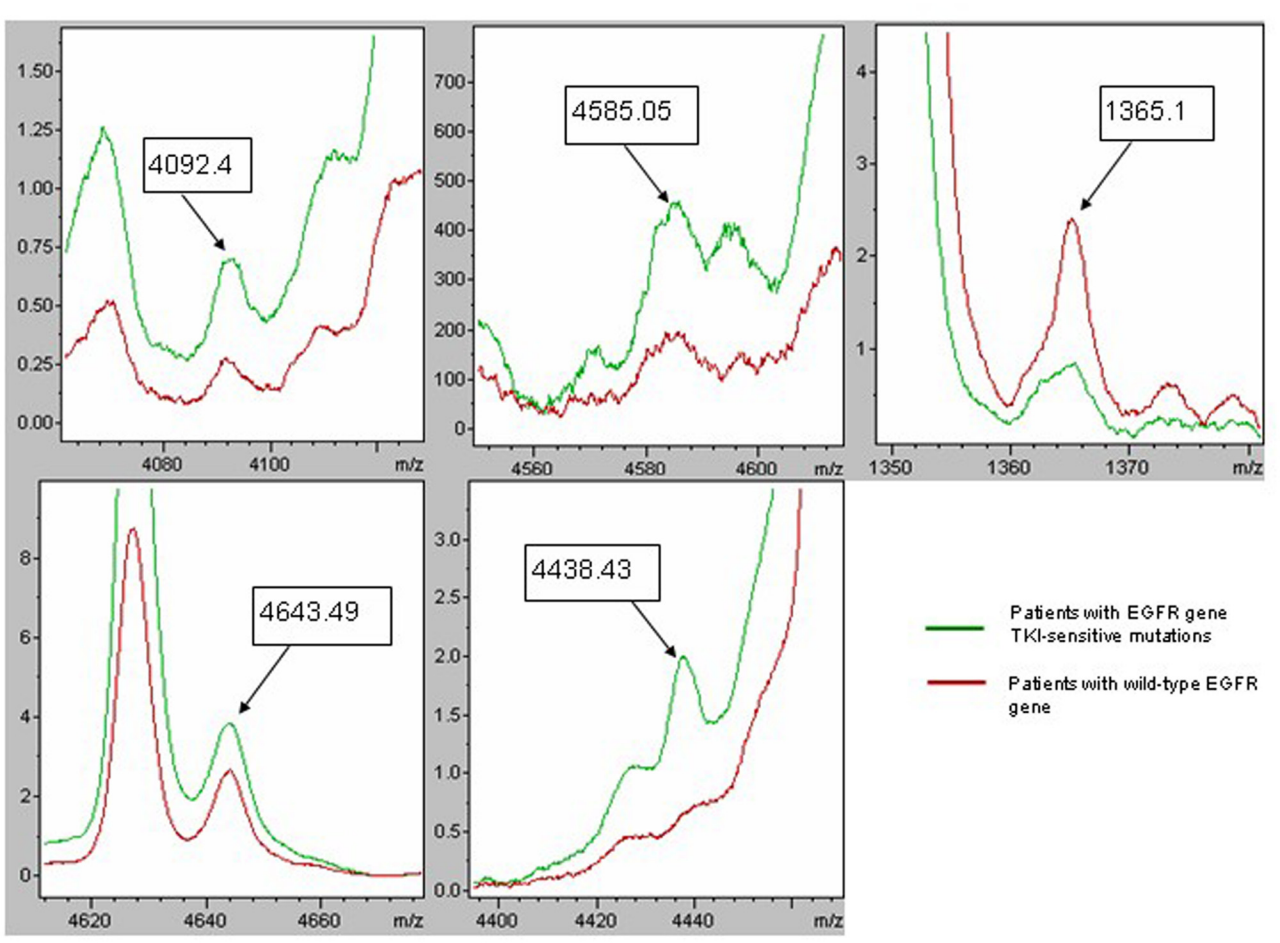
ClinProTools image showing the average intensity, in arbitrary units, of five peptides composing the classifier in patients with *EGFR* gene TKI-sensitive mutations and wild-type *EGFR* genes.

Three algorithms, GA (optimized by adjusting the number of neighbors for a k-nearest neighbor classification), SNN and QC, were applied for classification model construction using spectral data from the training group generated by MALDI-TOF-MS. The recognition capability and cross-validation of the models are presented in Table 5, and the Model GA-7 (named classifier), which was composed of five peptide peaks with m/z 4092.4, 4585.05, 1365.1, 4643.49 and 4438.43, exhibited the best efficiency in separating samples from patients with *EGFR* gene TKI-sensitive mutations and samples from patients with wild-type *EGFR* genes, with a recognition capability of 93.32% and a cross-validation of 81.23% (Fig 2).

**Table 5:** The cross-validation and recognition capability of three algorithms used to classify patients with *EGFR* gene TKI-sensitive mutations and wild-type *EGFR* genes.

Algorithm	Model name	Cross-validation (%)	Recognition capability (%)
**GA**			
** Number of neighbors: 3**	GA-3	75.50	92.16
** Number of neighbors: 5**	GA-5	74.52	93.30
** Number of neighbors: 7**	GA-7	81.23	93.32
**SNN**	SNN	74.04	91.28
**QC**	QC	64.01	81.62

GA = genetic algorithm; SNN = supervised neural network; QC = quick classifier algorithm.

### Blinded test of the classifier in the validation group

The classifier was then validated in an independent validation group of 123 NSCLC patients in a blinded test (Table 6). Three of the 123 samples yielded unclassifiable spectra (i.e., one sample was from a patient with *EGFR* gene TKI-sensitive mutation, and two were from patients with wild-type *EGFR* genes, as confirmed by ARMS in tumors). Among the 52 samples from patients with *EGFR* gene TKI-sensitive mutations confirmed by ARMS in tumors, 44 (84.6%) were labeled as “mutant” by the serum proteomic classifier; and among the 71 samples from patients with wild-type *EGFR* genes confirmed by ARMS in tumors, 55 (77.5%) were labeled as “wild” by the serum proteomic classifier, achieving an overall accuracy of 80.5%, with a sensitivity of 84.6% and a specificity of 77.5%, which indicated a high consistency between ARMS in tumors and the serum proteomic classifier in evaluating *EGFR* gene mutation status (P<0.001; Kappa value, 0.648; 3 patients with invalid spectra were excluded). However, of 52 samples from patients with *EGFR* gene TKI-sensitive mutations confirmed by ARMS in tumors, 7 (13.5%) were labeled as “wild” by the classifier; similarly, of 71 samples from patients with wild-type *EGFR* genes determined by ARMS in tumors, 14 (19.7%) were labeled as “mutant” by the classifier.

**Table 6:** Blind test results of the classifier in the validation group.

	Serum proteomic classifier		Invalid spectra	Total	Sensitivity (%)	Specificity (%)	Accuracy (%)
	Labeled as “mutant”	Labeled as “wild-type”					
**Determined by ARMS in tumors**							
***EGFR*-mutant**	44	7	1	52	84.6	77.5	80.5
***EGFR*-wild**	14	55	2	71			
** Total**	58	62	3	123*			

*P<0.001; Kappa value, 0.648; 3 patients with invalid spectra were excluded

### Correlation between *EGFR* gene TKI-sensitive mutations identified by the classifier and the therapeutic effect of EGFR-TKIs in the validation group

**Fig 3:**
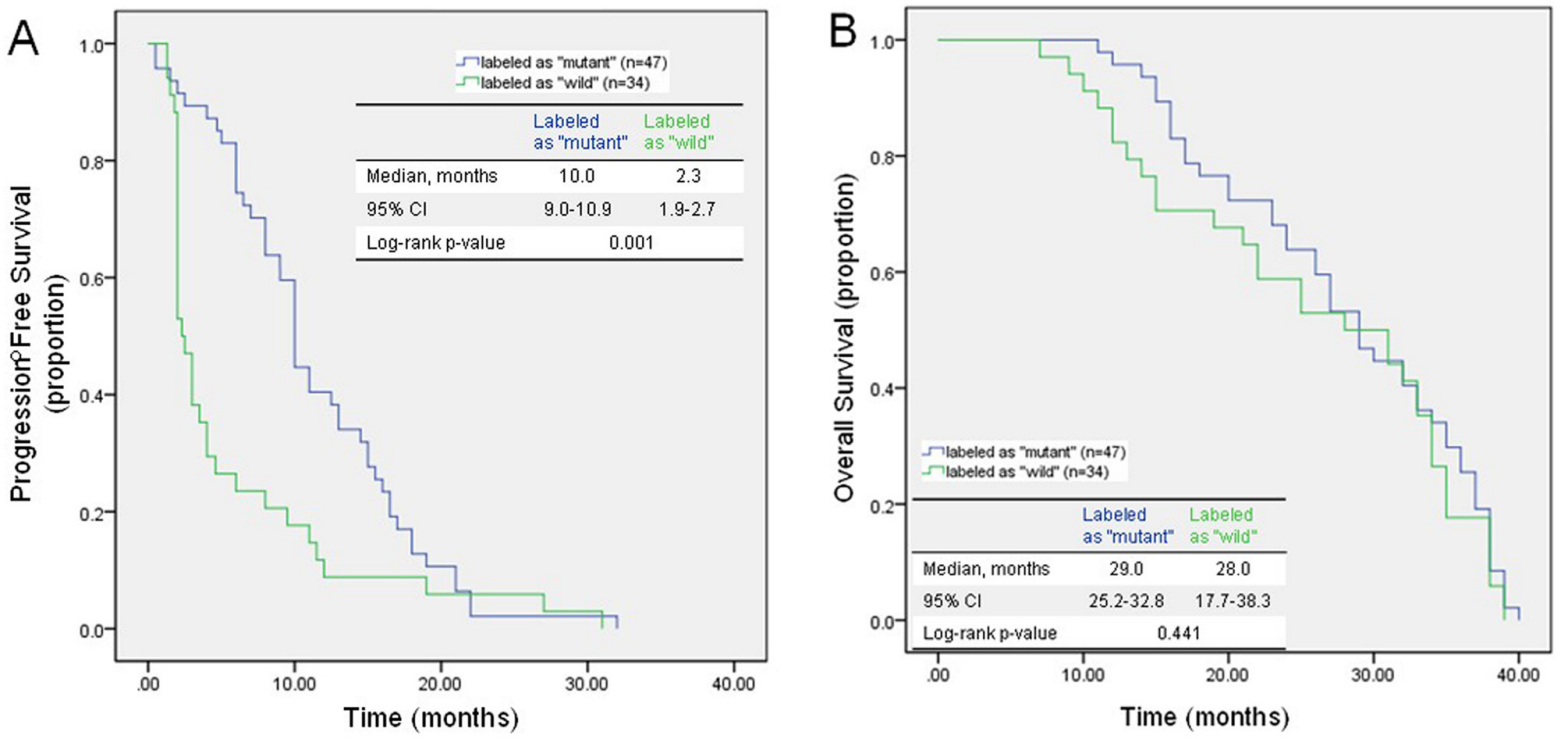
Kaplan-Meier plots of PFS (A) and OS (B) for 81 patients treated with EGFR-TKIs in the validation group. (A) PFS between patients whose matched samples were labeled as “mutant” (n = 47) and patients whose matched samples were labeled as “wild” (n = 34). (B) OS between patients whose matched samples were labeled as “mutant” (n = 47) and “wild” (n = 34).

In the validation group, three of the 123 samples yielded unclassifiable spectra, and the three corresponding patients were excluded from the analysis. Among the remaining 120 patients, 81 had measurable tumors and received EGFR-TKI treatment. The clinical and disease characteristics of these 81 patients are presented in Table 7, and the median follow-up time of these patients was 29.0 months (range, 7.0 to 40.0 months). Patients whose matched samples were labeled as “mutant” and “wild” by the classifier exhibited different tumor responses to EGFR-TKIs; these responses are listed in Table 8. Twenty-eight (59.6%) of 47 patients whose matched samples were labeled as “mutant” by the classifier and 3 (8.8%) of 34 patients whose matched samples were labeled as “wild” by the classifier exhibited an objective response (p<0.0001). Disease control was noted in 41 (87.2%) of 47 patients whose matched samples were labeled as “mutant” by the classifier and 12 (35.3%) of 34 patients whose matched samples were labeled as “wild” by the classifier (p<0.0001). Kaplan—Meier survival plots of PFS and OS for patients whose matched samples were labeled as “mutant” and “wild” by the classifier are shown in Fig 3. The median PFS time for patients whose matched samples were labeled as “mutant” and “wild” by the classifier were 10.0 months (95% CI, 9.0 to 10.9) and 2.3 months (95% CI, 1.9 to 2.7), respectively. Patients whose matched samples were labeled as “mutant” by the classifier had a significantly longer PFS than patients whose matched samples were labeled as “wild” by the classifier (p = 0.001, log-rank test, Fig 3A). Patients whose matched samples were labeled as “mutant” by the classifier had an OS time of 29.0 months (95% CI, 25.2 to 32.8) compared with 28.0 months (95% CI, 17.7 to 38.3) for the patients whose matched samples were labeled as “wild-type” by the classifier. There was no significant difference in OS between the two groups (p = 0.441, log-rank test, Fig 3B).

**Table 7:** Clinical and disease characteristics of patients enrolled in the analysis of EGFR-TKI therapeutic effects in the validation group.

Characteristics	Total (N = 81)	Labeled as “mutant” by the classifier (N = 47)	Labeled as “wild-type” by the classifier (N = 34)
**Age, y**			
** Mean**	55.1	55.1	55.2
** Standard deviation**	12.2	13.6	10.2
**Sex, No. (%)**			
** Male**	37(45.7)	19(40.4)	18(52.9)
** Female**	44(54.3)	28(59.6)	16(48.1)
**Smoking history, No. (%)**			
** Smoker**	34(42.0)	17(36.2)	17(50.0)
** Never smoker**	47(58.0)	30(63.8)	17(40.0)
**Histologic type, No. (%)**			
** ADC**	76(93.8)	45(95.8)	31(91.2)
** SCC**	3(3.7)	1(2.1)	2(5.9)
** Others**	2(2.5)	1(2.1)	1(2.9)
**Disease stage, No. (%)**			
** IIIB**	15(18.5)	8(17.0)	7(20.6)
** IV**	66(81.5)	39(83.0)	27(79.4)
**EGFR-TKI treatment, No. (%)**			
** First-line**	37(45.7)	29(61.7)	8(23.5)
** Second-line**	32(39.5)	15(31.9)	17(50.0)
** Third-line or greater**	12(14.8)	3(6.4)	9(26.5)
**EGFR-TKI, N (%)**			
** Gefitinib**	40(49.4)	24(51.1)	16(47.1)
** Erlotinib**	31(38.3)	17(36.2)	14(41.2)
** Icotinib**	10(12.3)	6(12.7)	4(11.7)
***EGFR* gene mutation status determined by ARMS, No. (%)**			
***E19del***	25(30.9)	21(44.7)	4 (11.8)
***L858R***	19(23.5)	16(34.0)	3(8.8)
***G719X***	3(3.7)	2(4.3)	1(2.9)
***wild-type***	34(41.9)	8(17.0)	26(76.5)

**Table 8:** Tumor response in patients whose matched samples were labeled as “mutant” and “wild” by the classifier in the validation group.

Classification	Response				Total	ORR (%)	DCR (%)
	CR	PR	SD	PD			
**Labeled as “mutant”**	0	28	13	6	47	59.6	87.2
**Labeled as “wild”**	0	3	9	22	34	8.8	35.3
**Total**	0	31	22	28	81		
**P value**						<0.001	<0.001

CR = complete response; PR = partial response; SD = stable disease; PD = progressive disease.

## Discussion

The assessment of *EGFR* gene mutation status in tumor tissue has important predictive value and can be used to select therapies for the treatment of NSCLC. Many patients with advanced and metastatic NSCLC are diagnosed with small biopsies or by fine needle aspiration of tumors, which often yields insufficient DNA for evaluating *EGFR* gene mutation status. Noninvasive approaches of evaluating *EGFR* gene mutation status using substitutes for tumor tissues would be of value for patients in whom sufficient tumor tissue is not available [16]. Table 9 provides details on the various methods used in previous reports to detect *EGFR* gene mutations in serum/plasma samples [3, 12–18]. Depending on the technique, the concordance between *EGFR* gene mutation status in tumor and plasma/serum samples ranges from 66% to 100%, with the highest correlation index being reported for denaturing high-performance chromatography [3] and mutant-enriched PCR [13]. However, these methods of assessing *EGFR* gene mutation status in plasma or serum samples are not widely used to guide EGFR-TKI therapy in clinical practice because of their inferior sensitivity when compared with findings from tumor tissue or the fact that studies examining these methods have utilized small sample sizes. In this study, we found that among 123 patients in the validation group, assessments of *EGFR* gene mutation status using serum proteomic classifiers yielded results that were concordant with the results of ARMS in tumors in 80.5% of the cases, with a high sensitivity of 84.6%.

**Table 9:** Methods used in selected previous reports to detect *EGFR* gene mutations in plasma and serum samples of lung cancer patients.

	Assay design	n	Sensitivity	Specificity	Sample
Bai[3]	Denaturing high-performance liquid chromatography	230	82%	90%	Plasma
Yung[12]	Microfiuidics digital PCR	35	92%	100%	Plasma
He[13]	Mutant-enriched PCR	18	100%	89%	Plasma
Jian[14]	Lightcycler PCR with Taqman-MGB probes	88	n.a.	n.a.	Plasma
Liu[15]	Scorpion-amplification refractory mutation system	86	67.5%	100%	Plasma
Brevet[16]	Mass spectrometry genotyping	31	44.4%	84.6%	Plasma
Kimura[17]	PCR + Direct sequencing	12	66%	63/71%^a^	Serum
Kimura[18]	Scorpion-amplification refractory mutation system	42	85%	94%	Serum

n.a.: Sensitivity and specificity are not available because of a lack of correlation with the primary matched tumors.

^a^: Before/after treatment.

However, we did find *EGFR* gene mutations that were successfully identified by only one method (i.e., serum proteomic classifier or ARMS in tumors) in 17.1% of patients in the validation group (i.e., 11.4% [14/123] by the serum proteomic classifier only, 5.7% [7/123] by ARMS in tumors only). It is important to note that cases in which test results were inconsistent between the serum proteomic classifier and ARMS in tumors cannot be considered as conventional “false-negatives” or “false-positives.” One possible explanation for this inconsistency in the determination of mutational status is heterogeneity of genetic abnormalities in the tumors. In such instances, tumor biopsy specimens might not carry the *EGFR* gene mutations identified by the serum proteomic classifier because these classifier-constituting peptides/proteins related to *EGFR* gene mutation status could be derived from different parts of the tumor. The lower tumor cell content in some of the tumors might also contribute to the lack of detectable mutations. Similarly, either there is little or no classifier-constituting peptides/proteins related to *EGFR* gene mutation status being shed in the blood in a given case, or the quantity of peptides/proteins in the serum is affected by certain conditions, such as inflammation, the classification of *EGFR* gene mutation status based on serum proteomic profiling might be impeded despite the presence of mutations in tumors.

EGFR encoded by the wild-type *EGFR* gene is a transmembrane tyrosine kinase receptor with a molecular weight of 170 kDa. The difference between EGFR encoded by *EGFR* gene with TKI-sensitive mutations and EGFR encoded by wild-type *EGFR* gene is that the former harbors activating tyrosine kinase domain. These two EGFRs should have similar molecular weights. Due to the high molecular weight, it is important to note that the MALDI-TOF-MS described in this study is neither suited for directly detecting EGFR encoded by *EGFR* gene with TKI-sensitive mutations nor EGFR encoded by wild-type *EGFR* gene because the typical observable mass range is 800–10000 Da. Instead, we detected the differential peptide/protein profiles between EGFR encoded by *EGFR* gene with TKI-sensitive mutations and EGFR encoded by wild-type *EGFR* gene. The identities of the constituting peptides/proteins are unknown at present; it is possible that they are unknown co-expressed peptides/proteins with low molecular weights involved, or that we detected fragments of EGFR or other high molecular weight proteins, such as proteins from the EGFR signaling pathway [29]. It is well known that tumor cell dissemination and apoptotic processes in tumors and at tumor-tissue boundaries involve changes in the proteolytic activities of a series of different proteases that may lead to the formation of protein fragments, thus providing a strong correlation with tumor tissue, and that as well serve as a basis for tumor differentiation and prognosis [29–32]. In agreement with this assumption, the proteins that have been identified thus far from blood samples by MALDI-TOF-MS have largely been degradation products of larger proteins [29, 33–36].

We also analyzed the potential implications of *EGFR* gene mutation status, as identified by the serum proteomic classifier, for predicting clinical outcomes in patients with NSCLC who received EGFR-TKIs. Our findings of a correlation between *EGFR* gene mutations identified by the classifier and tumor response to EGFR-TKI treatment and such treatment’s lack of impact on OS were also consistent with previous studies in which *EGFR* gene mutation status was tested in tumor tissue [4–8]. In patients treated with EGFR-TKIs in the validation group, 59.6% of the patients whose matched samples were labeled as “mutant” responded to EGFR-TKIs, whereas 8.8% of the patients whose matched samples were labeled as “wild” also responded. Although no difference in OS was observed between patients whose matched samples were labeled as “mutant” and “wild”, patients whose matched samples were labeled as “mutant” had significantly longer PFS after EGFR-TKI treatment, which suggests that these patients might have benefitted from the treatment. It should be noted that our study was not specifically designed to test EGFR-TKI treatment and that many patients received other chemotherapeutic agents, which makes data interpretation difficult. Additional clinical studies with specifically defined treatment regimens and larger sample sizes are necessary.

Tumor-based assays require well-preserved biopsy material, are technically difficult, incur substantial costs, and have a slow turnaround time. By contrast, the MALDI-TOF-MS method that we have described can be performed using less than 1 μl of pretreatment serum. Additionally, this method is inexpensive and rapid, and it can easily be fully automated. In our study, the assessment of *EGFR* gene mutation status using the serum proteomic classifier produced results that were not completely consistent with those obtained with ARMS in tumors. However, the inability to obtain primary tumor tissues, particularly through repeated biopsies, from patients with advanced-stage lung cancer makes the use of a serum proteomic classifier for analysis of *EGFR* gene mutation status clinically important given the high sensitivity (84.6%) of the technique and the favorable response to EGFR-TKIs in patients whose matched samples were labeled as “mutant” by the serum proteomic classifier.

One limitation of our analysis is the inability of the serum proteomic classifier to precisely determine the type of *EGFR* gene TKI-sensitive mutation, such as *exon 19* deletion (*E19del [LREA deletion]*) and *exon 21* mutation (*L858R*). Several studies have demonstrated that patients with an *exon 19* deletion experienced, on average, longer PFS and OS than those with an *L858R* mutation after first-line EGFR-TKI treatment for advanced non-small cell lung cancer [37, 38], indicating the clinical significance of the type of *EGFR* gene TKI-sensitive mutation. Therefore, our serum proteomic classifier must be modified to enable it to determine the type of *EGFR* gene TKI-sensitive mutation. Another limitation is the unknown biology underlying the correlation of these features with *EGFR* gene mutation status. Identification and analysis of the informative peaks might lead to important insights into the mechanisms underlying the correlation, and these studies are underway.

In conclusion, in this study, we detected differences in serum peptides/proteins between patients with *EGFR* gene TKI-sensitive mutations and patients with wild-type *EGFR* genes; based on these differences, a classification algorithm was developed for the analysis of *EGFR* gene mutation status. Furthermore, *EGFR* gene mutation status, as determined by the serum proteomic classifier, may be predictive of the response to EGFR-TKIs. All of the above provide evidence to suggest that a serum proteomic classifier may be used instead of tumor tissue for analysis of *EGFR* gene mutation status in NSCLC. It will be important to validate these findings and determine the value of the assay in predicting patients’ responses to TKIs in randomized trials with larger cohorts.

## References

[1] Siegel R, Ma J, Zou Z, Jemal A. Cancer statistics, 2014. CA Cancer J Clin. 2014 Jan-Feb;64(1):9–29. 10.3322/caac.21208 24399786

[2] Govindan R, Page N, Morgensztern D, Read W, Tierney R, Vlahiotis A, et al Changing epidemiology of small-cell lung cancer in the United States over the last 30 years: Analysis of the surveillance, epidemiologic, and end results database. 2006 10 1;24(28):4539–44. 17008692 10.1200/JCO.2005.04.4859

[3] Bai H, Mao L, Wang HS, Zhao J, Yang L, An TT, et al *Epidermal growth factor receptor* mutations in plasma DNA samples predict tumor response in Chinese patients with stages IIIB to IV non-small-cell lung cancer. J Clin Oncol. 2009 6 1;27(16):2653–9. 10.1200/JCO.2008.17.3930 19414683

[4] Fukuoka M, Wu YL, Thongprasert S, Sunpaweravong P, Leong SS, Sriuranpong V, et al Biomarker analyses and final overall survival results from a phase III, randomized, open-label, first-line study of geftinib versus carboplatin/paclitaxel in clinically selected patients with advanced non-small-cell lung cancer in Asia (IPASS). J Clin Oncol. 2011 7 20;29(21):2866–74. 10.1200/JCO.2010.33.4235 21670455

[5] Kim ES, Hirsh V, Mok T, Socinski MA, Gervais R, Wu YL, et al Geftinib versus docetaxel in previously treated non-small-cell lung cancer (INTEREST): a randomized phase III trial. Lancet. 2008 11 22;372(9652):1809–18. 10.1016/S0140-6736(08)61758-4 19027483

[6] Ciuleanu T, Stelmakh L, Cicenas S, Miliauskas S, Grigorescu AC, Hillenbach C, et al Efficacy and safety of erlotinib versus chemotherapy in second-line treatment of patients with advanced, non-small-cell lung cancer with poor prognosis (TITAN): a randomised multicentre, open-label, phase 3 study. Lancet Oncol. 2012 3;13(3):300–8. 10.1016/S1470-2045(11)70385-0 22277837

[7] Kawaguchi T, Ando M, Asami K, Okano Y, Fukuda M, Nakagawa H, et al Randomized phase III trial of erlotinib versus docetaxel as second- or third-line therapy in patients with advanced non-small-cell lung cancer: Docetaxel and Erlotinib Lung Cancer Trial (DELTA). J Clin Oncol. 2014 6 20;32(18):1902–8. 10.1200/JCO.2013.52.4694 24841974

[8] Shi Y, Zhang L, Liu X, Zhou C, Zhang L, Zhang S, et al Icotinib versus gefitinib in previously treated advanced non-small-cell lung cancer (ICOGEN): a randomised, double-blind phase 3 non-inferiority trial. Lancet Oncol. 2013 9;14(10):953–61. 10.1016/S1470-2045(13)70355-3 23948351

[9] Paez JG, Janne PA, Lee JC, Tracy S, Greulich H, Gabriel S, et al *EGFR* mutations in lung cancer: Correlation with clinical response to gefitinib therapy. Science. 2004 6 4;304(5676):1497–500. 15118125 10.1126/science.1099314

[10] Lynch TJ, Bell DW, Sordella R, Gurubhagavatula S, Okimoto RA, Brannigan BW, et al Activating mutations in the *epidermal growth factor receptor* underlying responsiveness of non—small-cell lung cancer to gefitinib. N Engl J Med. 2004 5 20;350(21):2129–39. 15118073 10.1056/NEJMoa040938

[11] Pao W, Miller V, Zakowski M, Doherty J, Politi K, Sarkaria I, et al *EGF receptor* gene mutations are common in lung cancers from “never smokers” and are associated with sensitivity of tumors to gefitinib and erlotinib. Proc Natl Acad Sci U S A. 2004 9 7;101(36):13306–11. 15329413 10.1073/pnas.0405220101 PMC516528

[12] Yung TK, Chan KC, Mok TS, Tong J, To KF, Lo YM. Single-molecule detection of *epidermal growth factor receptor* mutations in plasma by microfiuidics digital PCR in non-small cell lung cancer patients. Clin Cancer Res. 2009 3 15;15(6):2076–84. 10.1158/1078-0432.CCR-08-2622 19276259

[13] He C, Liu M, Zhou C, Zhang J, Ouyang M, Zhong N, et al Detection of *epidermal growth factor receptor* mutations in plasma by mutant-enriched PCR assay for prediction of the response to gefitinib in patients with non-small-cell lung cancer. Int J Cancer. 2009 11 15;125(10):2393–9. 10.1002/ijc.24653 19530244

[14] Jian G, Songwen Z, Ling Z, Qinfang D, Jie Z, Liang T, et al Prediction of *epidermal growth factor receptor* mutations in the plasma/pleural effusion to efficacy of gefitinib treatment in advanced non-small cell lung cancer. J Cancer Res Clin Oncol. 2010 9;136(9):1341–7. 10.1007/s00432-010-0785-z 20155428 PMC11828193

[15] Liu X, Lu Y, Zhu G, Lei Y, Zheng L, Qin H, et al The diagnostic accuracy of pleural effusion and plasma samples versus tumor tissue for detection of *EGFR* mutation in patients with advanced non-small cell lung cancer: comparison of methodologies. J Clin Pathol. 2013 12;66(12):1065–9. 10.1136/jclinpath-2013-201728 23888061 PMC3841772

[16] Brevet M, Johnson ML, Azzoli CG, Ladanyi M. Detection of *EGFR* mutations in plasma DNA from lung cancer patients by mass spectrometry genotyping is predictive of tumor *EGFR* status and response to EGFR inhibitors. Lung Cancer. 2011 7;73(1):96–102. 10.1016/j.lungcan.2010.10.014 21130517 PMC3282180

[17] Kimura H, Kasahara K, Shibata K, Sone T, Yoshimoto A, Kita T, et al *EGFR* mutation of tumor and serum in gefitinib-treated patients with chemotherapy-naïve non-small cell lung cancer. J Thorac Oncol. 2006 3;1(3):260–7. 17409866 10.1016/s1556-0864(15)31577-x

[18] Kimura H, Suminoe M, Kasahara K, Sone T, Araya T, Tamori S, et al Evaluation of *epidermal growth factor receptor* mutation status in serum DNA as a predictor of response to gefitinib (IRESSA). Br J Cancer. 2007 9 17;97(6):778–84. 17848912 10.1038/sj.bjc.6603949 PMC2360394

[19] Yanagisawa K, Shyr Y, Xu BJ, Massion PP, Larsen PH, White BC, et al Proteomic patterns of tumor subsets in non-small-cell lung cancer. Lancet. 2003 8 9;362(9382):433–9. 12927430 10.1016/S0140-6736(03)14068-8

[20] Pérez V, Ibernón M, López D, Pastor MC, Navarro M, Navarro-Muñoz M, et al Urinary peptide profiling to differentiate between minimal change disease and focal segmental glomerulosclerosis. PLoS One. 2014 1 30;9(1):e87731 10.1371/journal.pone.0087731 24498182 PMC3907468

[21] Wang L, Liu HY, Shi HH, Lang JH, Sun W. Urine peptide patterns for non-invasive diagnosis of endometriosis: a preliminary prospective study. Eur J Obstet Gynecol Reprod Biol. 2014 6;177:23–8. 10.1016/j.ejogrb.2014.03.011 24694773

[22] Hong WX, Liu W, Zhang Y, Huang P, Yang X, Ren X, et al Identification of serum biomarkers for occupational medicamentosa-like dermatitis induced by trichloroethylene using mass spectrometry. Toxicol Appl Pharmacol. 2013 11 15;273(1):121–9. 10.1016/j.taap.2013.08.014 23994554

[23] He A, Bai J, Huang C, Yang J, Zhang W, Wang J, et al Detection of serum tumor markers in multiple myeloma using the CLINPROT system. Int J Hematol. 2012 6;95(6):668–74. 10.1007/s12185-012-1080-3 22539364

[24] Shin S1, Cazares L, Schneider H, Mitchell S, Laronga C, Semmes OJ, et al Serum biomarkers to differentiate benign and malignant mammographic lesions. J Am Coll Surg. 2007 5;204(5):1065–71; discussion 1071–3. 17481542 10.1016/j.jamcollsurg.2007.01.036

[25] Yang J, Song YC, Song TS, Hu XY, Guo YM, Li ZF, et al Identification of novel low molecular weight serum peptidome biomarkers for non-small cell lung cancer (NSCLC). J Clin Lab Anal. 2012 5;26(3):148–54. 10.1002/jcla.21502 22628229 PMC6807414

[26] Taguchi F, Solomon B, Gregorc V, Roder H, Gray R, Kasahara K, et al Mass spectrometry to classify non-small-cell lung cancer patients for clinical outcome after treatment with epidermal growth factor receptor tyrosine kinase inhibitors: a multicohort cross-institutional study. J Natl Cancer Inst. 2007 6 6;99(11):838–46. 17551144 10.1093/jnci/djk195

[27] Wu X, Liang W, Hou X, Lin Z, Zhao H, Huang Y, et al Serum proteomic study on EGFR-TKIs target treatment for patients with NSCLC. Onco Targets Ther. 2013 10 21;6:1481–91. 10.2147/OTT.S51887 24204163 PMC3818102

[28] Gregorc V, Novello S, Lazzari C, Barni S, Aieta M, Mencoboni M, et al Predictive value of a proteomic signature in patients with non-small-cell lung cancer treated with second-line erlotinib or chemotherapy (PROSE): a biomarker-stratified, randomised phase 3 trial. Lancet Oncol. 2014 6;15(7):713–21. 10.1016/S1470-2045(14)70162-7 24831979

[29] Rauser S, Marquardt C, Balluff B, Deininger SO, Albers C, Belau E, et al Classification of *HER2* receptor status in breast cancer tissues by MALDI imaging mass spectrometry. J Proteome Res. 2010 4 5;9(4):1854–63. 10.1021/pr901008d 20170166

[30] Annecke K, Schmitt M, Euler U, Zerm M, Paepke D, Paepke S, et al uPA and PAI-1in breast cancer: review of their clinical utility and current validation in the prospective NNBC-3 trial. Adv Clin Chem. 2008;45:31–45. 18429492 10.1016/s0065-2423(07)00002-9

[31] Hojilla CV, Wood GA, Khokha R. Infiammation and breast cancer: metalloproteinases as common effectors of infiammation and extracellular matrix breakdown in breast cancer. Breast Cancer Res. 2008;10(2):205 10.1186/bcr1980 18394187 PMC2397522

[32] Lopez-Otin C, Matrisian L. Emerging roles of proteases in tumour suppression. Nat Rev Cancer. 2007 10;7(10):800–8. 17851543 10.1038/nrc2228

[33] Milan E, Lazzari C, Anand S, Floriani I, Torri V, Sorlini C, et al SAA1 is over-expressed in plasma of non-small cell lung cancer patients with poor outcome after treatment with epidermal growth factor receptor tyrosine-kinase inhibitors. J Proteomics. 2012 12 5;76 Spec No.:91–101. 10.1016/j.jprot.2012.06.022 22771314

[34] Liu J, Yin L, Dong H, Xu E, Zhang L, Qiao Y, et al Decreased serum levels of nucleolin protein fragment, as analyzed by bead-based proteomic technology, in multiple sclerosis patients compared to controls. J Neuroimmunol. 2012 9 15;250(1–2):71–6. 10.1016/j.jneuroim.2012.04.021 22633274

[35] Tang W, Shi YQ, Zou JJ, Chen XF, Zheng JY, Zhao SW, et al Serum biomarker of diabetic peripheral neuropathy indentified by differential proteomics. Front Biosci (Landmark Ed). 2011 6 1;16:2671–81. 21622202 10.2741/3879

[36] Xi L, Junjian Z, Yumin L, Yunwen L, Hongbin W. Serum biomarkers of vascular cognitive impairment evaluated by bead-based proteomic technology. Neurosci Lett. 2009 9 29;463(1):6–11. 10.1016/j.neulet.2009.07.056 19631719

[37] Zhang Y, Sheng J, Kang S, Fang W, Yan Y, Hu Z, et al Patients with *exon 19* deletion were associated with longer progression-free survival compared to those with *L858R* mutation after first-line EGFR-TKIs for advanced non-small cell lung cancer: a meta-analysis. PLoS One. 2014 9 15;9(9):e107161 10.1371/journal.pone.0107161 25222496 PMC4164616

[38] Wu YL, Zhou C, Hu CP, Feng J, Lu S, Huang Y, et al Afatinib versus cisplatin plus gemcitabine for first-line treatment of Asian patients with advanced non-small-cell lung cancer harbouring *EGFR* mutations (LUX-Lung 6): an open-label, randomised phase 3 trial. Lancet Oncol. 2014 2;15(2):213–22. 10.1016/S1470-2045(13)70604-1 24439929

